# The relationship between dispositional empathy and prefrontal cortical functioning in patients with frontal lobe epilepsy

**DOI:** 10.12669/pjms.331.11742

**Published:** 2017

**Authors:** Amara Gul, Hira Ahmad

**Affiliations:** 1 Amara Gul, PhD Department of Applied Psychology, The Islamia University of Bahawalpur, Bahawalpur, Pakistan; 2 Hira Ahmad, MPhil Clinical Psychology, Department of Applied Psychology, The Islamia University of Bahawalpur, Bahawalpur, Pakistan

**Keywords:** Prefrontal cortical functioning, Cognition, Empathy, Executive functions, Frontal lobe epilepsy

## Abstract

**Background & Objective:**

Rehabilitation focuses brain-behavior relationship which highlights interaction between psychological and neurobiological factors for better patient care. There is a missing link in the literature about socio-cognitive aspects of frontal lobe epilepsy. Our objective was to examine prefrontal cortical functioning (PCF) and empathic abilities in patients with frontal lobe epilepsy (FLE). Further, we analyzed whether any relationship between components of dispositional empathy and PCF exists in patients with FLE.

**Methods:**

The study was designed in an experimental paradigm. Sixty patients with FLE were recruited from Sheikh Zayed and Jinnah hospital, Pakistan. Sixty healthy individuals in response to an advertisement took part in the study as control subjects. Participants completed interpersonal reactivity index. Following they performed clock drawing test and word-color identification task switching experiment.

**Result:**

Patients with FLE demonstrated weaker PCF (i.e., cognitive flexibility and executive function) as compared to healthy control subjects. Patients with FLE scored lesser on cognitive empathy as compared to healthy control subjects. On contrary, there was no significant difference between patient and control group on affective empathy. Cognitive not affective empathy was potential predictor of PCF.

**Conclusion:**

Cognitive empathy is a significant marker of prefrontal cortical functioning (PCF) in FLE. Higher cognitive empathy would lead to efficient PCF.

## INTRODUCTION

Frontal lobe epilepsy (FLE) is characterized by frequent seizures of the frontal lobe. In cerebral cortex, frontal lobes encompass various dopamine-sensitive neurons which are responsible for prefrontal cortical functioning (i.e., executive function and cognitive flexibility). Deficit in executive function (EF) has been observed in patients with FLE^1^ whereas cognitive flexibility (CF) is scarcely examined. CF requires rapid adaptation of new task-set when the situation changes. This involves shift of attention and action according to the switched (new) task. An efficient switching reflects the execution of control processes that reconfigure mental resources when the task is alternated. The differential performance between switch and no-switch situations with reference to speed and accuracy is known as switch cost (SC) that arises from executive control processes. These processes are required to reconfigure the cognitive system to implement new task-set when the task switches.^2^ Neurocognitive studies demonstrated that executive control processes operate through prefrontal cortex (PC) whereas lesions of PC interfere with the normal execution of control processes.^3^

Socio-cognitive functioning of patients with FLE is also disturbed.^4^ Complex interactions between psychosocial and neurobiological functioning determine patient care.^5^ Epilepsy has adverse effects on empathic abilities.^6^ Empathy is the capacity to process emotions of other people. This ability influence social relationships in positive manner, for instance being more compassionate or sympathetic. On the other hand, deficient empathy has adverse social consequences because it will lead to negative behaviors (e.g., resentment, aggression). Thus the ability to share another’s internal states and explicitly considering those states is known as dispositional empathy (DE) which is typically divided into cognitive and affective components.^7^ Cognitive empathy (CE) is the capacity to understand perspective or mental state of others. It is taken as synonymous term for theory of mind. CE is a conscious drive to recognize and accurate understanding of emotions of other people. People understand others by a cognitive function known as mentalizing that activates PC.^8^ Neuropsychological studies have shown that patients with prefrontal damage perform worse on social cognition tasks.^9^ On the other hand, Affective empathy (AE) is the capacity to respond in accordance with the emotional state of others.^10^ This is an automatic tendency to indirectly share emotions and feelings of other people. In order to work efficiently in daily routine, one has to control AE for better self-management and unbiased decision making.

Human brain responds differently when cognitive and affective empathy is activated because distinct brain areas are involved in these dissociated capacities. CE is deliberate and involve higher order cognitive functions such as abstraction and inference. It is modulated by regions of the prefrontal cortex. In contrast, AE is controlled by emotion centers of the brain (e.g., amygdala) and mirror neurons. Lower CE and AE correlate with smaller volumes in right frontal and limbic regions.^11^ CE is modulated by ventromedial PC whereas AE involves mid cingulate cortex, anterior insula, inferior frontal gyrus.^12^ Lesions of the amygdala and inferior frontal cortex disrupt AE, in contrast lesions of the medial PC disturb CE.^13^ Cognitive processes modulate empathic experience and rely on same neural substrate of empathic experience in lateral and medial PC.^14^ Functional magnetic resonance imaging studies have shown that PC is involved in constructing cognitive mechanisms during emotion processing.^15^ Social cognition is well studied phenomenon in patients with FLE, but there is no study that examined components of DE and prefrontal cortical functioning (PCF) in connection with FLE. Given that brain regions involved in PCF and empathic abilities overlap, there is a possibility that these parameters interact resulting in deficient PCF and impaired CE. Moreover, components of CE might be a potential marker of PCF deficits in patients with FLE. None of the previous studies regarding impacts of FLE on cognition and emotion processing have examined relationship between PCF and components of empathic abilities in FLE. Thus, there is a gap in the literature to provide understanding whether these variables interact in patients with FLE. Objectives for the current study were as follows:

1. To compare prefrontal cognitive functioning (i.e., executive function and cognitive flexibility) between patients with FLE and healthy control individuals.
2. To examine components of empathy (i.e., cognitive and affective) in patients with FLE and healthy control individuals.
3. To assess components of empathy as potential predictors of PCF.

### Hypotheses

Following hypotheses were formulated for the present study to be examined.

1. In contrast to healthy control individuals, patients with FLE would exhibit deficits in PCF (i.e., executive function and cognitive flexibility).
2. Patients with FLE would show impairments in CE and AE. On contrary, healthy control individuals would show efficient task switching.
3. Components of DE would predict PCF.

## METHODS

Sixty patients with FLE were included in the study at Sheikh Zayed and Jinnah Hospital, Pakistan from September 2013 to December 2014. The inclusion criterion were as follows: (i) ictal or interictal EEG evidence and consistent seizure semiology of clear onset in the frontal lobe (ii) MRI evidence of epileptogenic lesions of the frontal lobe (iii) normal intellectual function as measured by Standard Progressive Matrices.^16^ Patients were excluded from the sample in case having dysfunction or epileptogenic focus outside the frontal regions (ii) history or present psychiatric illness (iii) below average intellectual functioning. All patients were on antiepileptic medication. Lesion sites were determined by MRI or EEG examination. Sixty healthy individuals (control group) were contacted from the local community through an advertisement. Individuals having an average intellectual functioning and with no history of psychiatric, neurological illness and medication use were included in the sample. Subject groups were matched on demographic variables (Table-I).

**Table-I:** Characteristics of Patient and Control Group.

	Patients (n=60)	Controls (n=60)	

Gender (M/F)	30/30	30/30	

	M (SD)	M (SD)	t, p
Age (Years)	28.70 (1.39)	28.83 (1.98)	
Education (Years)	12.83 (1.36)	12.76 (1.25)	
Intellectual Function	50 (1.24)	56 (1.76)	
Age at epilepsy onset (Years)	13.23 (1.78)	Nil	
Localization of Abnormality			
Dorsolateral	15	Nil	
Prefrontal cortex	25	Nil	
Medial	10	Nil	
Orbitofrontal foci	10	Nil	
Cognitive empathy	42.65 (2.25)	43.05 (2.08)	t(59)=4.18, p<0.001
Affective empathy	46.26 (1.60)	46.78 (1.72)	t(59)=1.50, p=0.138
Clock drawing test	3.30 (1.21)	8.16 (1.15)	t(59)=21.52, p<0.001

### Materials

### Interpersonal Reactivity Index (IRI): Cognitive and Affective Empathy

IRI is a self-report measure of DE which provides index for subscales of DE: CE and AE. IRI has good psychometric properties.^17^

### Assessment of Prefrontal Cortical Functioning

### Clock Drawing Test: Executive Function

Participants were presented with a pre-drawn circle and were instructed to place numbers so that it looks like a clock. Further, they were asked to place hands of the clock to read “10 past eleven”. Clocks were scored by using 10 -point Sunderland method that takes into account the hand positioning. A score of 6 or more is considered normal on scale ranging from 0=very poor to 10=perfect.^18^

### Color-Word Identification Task Switching Experiment: Cognitive Flexibility

Task switching experiment was designed in E-prime software^19^ and was presented to the participants on laptop screen. Stimuli consisted of eight colored words. Out of eight, four words: yellow, green, red and blue. These words were presented in the congruent ink whereas same four words were presented in incongruent ink. Stimuli were same for both tasks and were cued by different backgrounds. There were 128 trials in the experiment.

### Procedure

The study was conducted in accordance with principles of Helsinki Declaration and was approved by the board of studies of the Islamia University of Bahawalpur, Pakistan. All participants signed an informed consent form. Participants completed IRI and clock drawing test, following this they performed task switching experiment.

## RESULTS

Results were computed using SPSS software (version 20). Group differences on CE, AE and Clock drawing test are shown in Table-I. Reaction times (RTs) were discarded above 2.5 standard deviations from each participants’ mean. SC (mean RTs switch minus no-switch trials) were calculated. To examine task switching data, mean RTs were submitted to a 2 x 2 repeated measures analysis of variance with trial (*switch* vs. *no-switch*) as within subject factors and group (*patients with FLE vs. healthy controls*) as between subject factor. The main effect of trial was significant *F* (1, 118) =1057.25, *p*<0.001, ηp2=.90. RTs were slower on switch (*M*=1651.00 milliseconds) than no-switch trials (*M*=927.54 milliseconds). The main effect of group was significant *F* (1, 118)=8.32, *p*<0.05, ηp2=.06, patients with FLE (*M*=1335.74) controls (*M*= 1242.76). There was a significant interaction between trial x group *F* (1, 118) =4.71, *p*<0.05, ηp2=.03. Patients with FLE performed slower on switch trials than control group *t* (59) =2.92, *p*<0.05. However, there was no significant difference on no-switch trials *t* (59)*=* 1.39, *p*=.169. Patients with FLE showed larger SC (*M*=771.73ms) than control group (*M*=675.10 milliseconds), *t* (59)*=* 2.13, *p*<0.05. Results of regression analysis to examine predictors of DE and SC are depicted in Table II and III.

**Table-II:** Linear Regression Analysis with Switch costs as Dependent and Components of DE as Independent Variables.

	β	t		
Cognitive empathy	0.40	4.81, p<0.001	R^2^ =0.19	F (2, 119) =14.04, p<0.001
Affective empathy	0.14	1.77, p=0.078		

**Table-III:** Linear Regression Analysis with Executive functions as Dependent and Components of DE as Independent Variables.

	β	t		
Cognitive empathy	0.24	2.67, p<0.001	R^2^ =0.05	F (2, 119) =3.58, p<0.05
Affective empathy	0.00	0.10, p=0.91		

## DISCUSSION

Present study was designed to investigate whether dispositional empathy have any influence on basic and higher order cognitive abilities. There were few important results: (i) patients with FLE showed marked deficits in components of DE (ii) Patients with FLE demonstrated impairment in PCF (iii) CE proved to be the only potential predictor of PCF. Results are in line with previous studies which showed deteriorated cognitive functioning and empathy in epilepsy.^3^-^4^,^7^,^20^ PC is controlled by interconnected neocortical areas which are responsible for nerve signal transmissions almost all over subcortical structures and sensory-motor systems.^21^ These connections are disrupted in FLE. Impaired empathic abilities are associated with reduced empathy-related brain response in amygdala and periaqueductal gray that is necessary for an empathic experience.^11^ The hypotheses that FLE would correlate with impairments in PCF and top-down control of behaviors was confirmed as patients with FLE showed weaker performance on cognitive flexibility, EF and cognitive empathy as compared to healthy controls. Frontal lobe plays crucial role in regulating higher order cognitions, emotions and behaviors. Dysfunctions of the frontal lobe are associated with weaker nerve signals that are sensitive to emotion-related and cognitive tasks,^22^ thus performance of patients with FLE and frontal lobe damage is comprised.^23^ Previous studies have shown that CE is correlated with empathy in patients with idiopathic generalized epilepsy.^24^

Findings of the present study demonstrated that CE but not AE predicted PCF. CE considers rationalization of behavior taking rules, norms, morality and social situations into account. In contrast, AE is uncontrolled emotional reaction aroused by the situational circumstances. CE served as a modulator for PCF. Higher CE is related with rapid PCF. Neural correlates related with higher order cognitions and empathy converge in bilateral temporal-parietal junction, dorsal medial PC, and middle temporal gyrus which form a shared network which is involved in social-cognitive processes.^25^ Deficient CE would lead to psychopathy and antisocial behavior.^26^ CE is essential to interpret social information and learning from emotion laden situations. Pathological conditions affecting frontal and orbital cortex in the form of lesions or psychiatric disorders (e.g., bipolar disorder) face difficulty in processing emotions.^27^,^28^ Cognitive theories highlight that CE involves several cognitive processes such as shifting attention to understand perspective of other people, role-taking, and responding to situations in non-egocentric manner.^29^

These results have implications for neuro-rehabilitation of patients with FLE. Cognitive rehabilitation programs must be designed with specific goals to improve empathic abilities. Such intervention programs can help patients to overcome difficulties related with EF and task switching in daily life.

### Limitations of the study

Small sample size. A larger sample size will increase the generalizability. Theory of mind tasks as an instrument will also provide a comprehensive picture of empathic abilities. Future studies must also focus cognitive behavioral therapies as an intervention to improve affective and cognitive facets of empathy in patients with FLE to prevent further cognitive deterioration.

## CONCLUSION

Impaired CE influences PCF in frontal lobe epilepsy. This can serve as an indicator for therapeutic intervention in patients with FLE. Early detection of compromised CE could inhibit further deterioration in PCF. Future studies must examine whether weakened CE could be improved with training.
